# Correction: Bisighini et al. Fabrication of Compliant and Transparent Hollow Cerebral Vascular Phantoms for In Vitro Studies Using 3D Printing and Spin–Dip Coating. *Materials* 2023, *16*, 166

**DOI:** 10.3390/ma17102405

**Published:** 2024-05-17

**Authors:** Beatrice Bisighini, Pierluigi Di Giovanni, Alba Scerrati, Federica Trovalusci, Silvia Vesco

**Affiliations:** 1 Mines Saint-Etienne, Université Lyon, Université Jean Monnet, Etablissement Français du Sang, INSERM, U1059 Sainbiose, Centre CIS, F-42023 Saint-Etienne, France; 2 Department of Enterprise Engineering, University Tor Vergata, Via del Politecnico 1, 00133 Rome, Italy; 3 Predisurge, 10 Rue Marius Patinaud, Grande Usine Creative 2, 42000 Saint-Etienne, France; 4 HSL s.r.l., Via dei Masadori 46, 38121 Trento, Italy; 5 Department of Translational Medicine, University of Ferrara, Via Luigi Borsari 46, 44121 Ferrara, Italy

In the original publication [1], there was a mistake in the caption for Table 2. The correct caption appears below: 

Table 2. SLA printing settings for water soluble printable resin.

A correction has been made to *2. Material and Methods*, *2.2. 3D Printing*, *2.2.2. Stereolithography*, *Paragraph 1*:

In SLA, a computer-controlled laser beam is focused onto a photo-polymerisable resin, and the full model is obtained by curing this liquid layer by layer. In this work, the 3D printer employed was a Zortrax Inkspire (Zortrax, Olsztyn, Poland), while a water-soluble resin was selected for this study. Its printing settings are reported in Table 2. The models printed by SLA needed to be UV-cured for 20 min at 35 °C before manipulations. The 3D printer employed had an XY resolution of 50 microns and a minimum layer thickness of 25 microns.

The authors state that the scientific conclusions are unaffected. This correction was approved by the Academic Editor. The original publication has also been updated.
